# Intravenous Albumin for Oedema in Children With Nephrotic Syndrome: A Systematic Review and Mapping of the Evidence Landscape

**DOI:** 10.7759/cureus.95142

**Published:** 2025-10-22

**Authors:** Sohaib B Nawaz, Muhammad Ashfaq Zafar

**Affiliations:** 1 Paediatrics and Child Health, School of Paediatrics, Yorkshire and Humber, GBR; 2 Paediatric Medicine, Scunthorpe General Hospital, Scunthorpe, GBR; 3 Paediatric Medicine, Mayo University Hospital, Castlebar, IRL

**Keywords:** albumin (alb), childhood nephrotic syndrome, nephrology disorders, nephrotic, paediatric disease, swelling (oedema), systematic mapping, systematic review

## Abstract

Nephrotic syndrome is a common glomerular disorder in childhood, often complicated by oedema, which contributes to morbidity, discomfort and prolonged hospitalisation. Intravenous (IV) albumin is frequently used with diuretics in children with resistant oedema, but its effectiveness and safety remain uncertain. Previous reviews are limited by adult populations, small sample sizes or a lack of quantitative synthesis. Our aim was to systematically evaluate the effectiveness and safety of IV albumin, with or without diuretics, versus no albumin in hospitalised children with nephrotic syndrome and oedema, and to map excluded-but-relevant studies to identify evidence gaps. We searched PubMed, Cochrane CENTRAL, Google Scholar and Europe PMC from January 1, 1990, to June 30, 2025, using MeSH terms and free-text keywords for nephrotic syndrome, albumin and paediatric populations. Reference lists of eligible studies and prior reviews were hand-searched. We included randomised controlled trials (RCTs), quasi-RCTs and comparative observational studies in children aged 1-18 years hospitalised with nephrotic syndrome and oedema. Studies without a comparator, non-paediatric populations, non-English publications or single-arm case series were excluded. Two reviewers independently screened records, extracted data and assessed risk of bias using the Cochrane Risk of Bias 2.0 tool (ROB 2) for RCTs. Due to the paucity of eligible studies and heterogeneity, outcomes were narratively synthesised. Excluded-but-relevant studies were mapped to identify evidence gaps. Out of 784 screened records, only one RCT met the inclusion criteria. Albumin plus furosemide significantly increased short-term urine output and weight reduction compared to furosemide alone, but effects were transient, and patient-centred outcomes were largely unassessed. Evidence mapping revealed multiple excluded studies with methodological limitations, inappropriate comparators or adult/mixed populations. Previous reviews corroborated the transient physiological effects but lacked paediatric-specific conclusions. We concluded that evidence supporting IV albumin use in hospitalised children with nephrotic syndrome is extremely limited. Albumin may provide short-term physiological benefit in select cases, but routine use is not justified. High-quality, adequately powered paediatric RCTs with standardised outcomes and systematic safety reporting are urgently needed to guide clinical practice.

## Introduction and background

Nephrotic syndrome is one of the most prevalent glomerular disorders in childhood, with an annual incidence ranging between one and seven per 100,000 children worldwide [1]. It is defined by the presence of heavy proteinuria, hypoalbuminaemia and oedema, often accompanied by hyperlipidaemia. Oedema is the most visible and distressing manifestation of the condition, with severity ranging from mild periorbital swelling to generalised anasarca [2]. In hospitalised children, oedema can cause significant morbidity, discomfort, delayed recovery and extended hospital stays [3].

The underlying mechanisms of oedema in nephrotic syndrome are multifactorial. Two dominant theories explain the pathogenesis of oedema [4-6]. The underfill hypothesis suggests that hypoalbuminaemia reduces plasma oncotic pressure, leading to intravascular volume depletion and secondary sodium and water retention via the renin-angiotensin-aldosterone system (RAAS). In contrast, the overfill hypothesis attributes oedema to primary renal sodium retention, driven by tubular dysfunction in the distal nephron [4-6]. Both mechanisms likely contribute to varying degrees depending on disease stage and severity [3].

Management of oedema in children with nephrotic syndrome typically involves fluid and salt restriction along with loop diuretics such as furosemide. However, diuretic resistance is commonly encountered, particularly in the setting of profound hypoalbuminaemia [7]. In such cases, intravenous (IV) albumin is frequently administered in combination with diuretics, with the rationale that albumin can temporarily increase plasma oncotic pressure, improve intravascular volume and enhance renal perfusion, thereby restoring the efficacy of loop diuretics [3,8].

This approach is widely used in clinical practice across many paediatric nephrology units [9,10]. However, its benefits remain uncertain. Some studies report short-term improvements in urine output and weight loss [11], but the effects appear transient unless remission of proteinuria is achieved. Importantly, albumin infusion is not without risk. Adverse events, including acute hypertension, hypokalaemia, hypernatraemia, fluid overload and respiratory distress, have been frequently documented [12]. In one retrospective study, hypertension requiring urgent management occurred in nearly half of albumin treatment episodes, and one child developed congestive heart failure [13]. Given its potential for harm and resource intensity, the use of IV albumin in this setting warrants a strong evidentiary foundation demonstrating both clinical benefit and safety.

However, the current literature does not provide the clarity required for paediatric clinical practice. Several previous systematic reviews have attempted to assess the role of intravenous albumin in oedematous or hypoalbuminaemic patients, but all have critical limitations that restrict their applicability to children with nephrotic syndrome. Elwell et al. (2003) conducted a narrative review that focused primarily on adult populations in critical care and cirrhotic settings. While they discussed pharmacological mechanisms, their findings were inconsistent and based on small studies, with limited relevance to paediatric nephrotic syndrome [7]. Kitsios et al. (2014) performed a meta-analysis of eight randomised controlled trials, largely in adults, and reported modest increases in urine output and sodium excretion at eight hours post-intervention but no sustained effects at 24 hours [14]. Their analysis excluded paediatric populations entirely. Hedin et al. (2022), although published in a paediatric nephrology journal, synthesised data from five studies, four of which were conducted in adults, and did not stratify findings by age group, making its conclusions difficult to extrapolate to children [3]. The Cochrane review by Ho et al. (2019) aimed to evaluate albumin therapy in nephrotic syndrome across all ages, but ultimately included only one paediatric study that was published in Korean and inaccessible to many readers [5]. Collectively, these reviews highlight a striking lack of focused, high-quality evidence to guide the use of IV albumin in hospitalised children with nephrotic syndrome and significant oedema, particularly in comparison to standard treatment without albumin.

This systematic review was designed to fill that gap. Following a registered protocol, it evaluates the effectiveness and safety of intravenous albumin, with or without diuretics, versus no albumin in hospitalised children aged 1-18 years with nephrotic syndrome and oedema. By focusing on comparative studies in this well-defined clinical context, the review aimed to support evidence-based decision-making and improve the management of a high-impact paediatric condition.

## Review

Methods and materials

Protocol and Registration

This review was conducted according to a predefined protocol registered with the International Prospective Register of Systematic Reviews (PROSPERO: CRD420251102387), on July 11, 2025, which is available at https://www.crd.york.ac.uk/PROSPERO/view/CRD420251102387. The reporting of this systematic review adheres to the Preferred Reporting Items for Systematic Reviews and Meta-Analyses (PRISMA) 2020 guidelines (Appendices).

Eligibility Criteria

Eligibility was defined using the Population, Intervention, Comparator, Outcomes, Study Designs and Timeframe (PICOST) framework, as shown in Table 1.

**Table 1:** Eligibility criteria RCT: randomised controlled trial

Criteria	Eligibility details
Population	Children aged 1-18 years with nephrotic syndrome and clinically evident oedema during hospitalisation.
Intervention	Intravenous albumin administered either alone or in combination with diuretics such as furosemide.
Comparator	Diuretics alone, placebo or supportive care without albumin.
Outcomes	Studies were eligible if they reported on at least one of the following: change in weight (primary), urine output (secondary), time to oedema resolution (secondary), length of hospital stay (secondary) or adverse events (primary).
Study Designs	We included RCTs, quasi-RCTs and comparative observational studies.
Timeframe	We screened studies reported from January 1, 1990, to June 30, 2025.

We excluded studies meeting any of the criteria shown in Table 2.

**Table 2:** Exclusion criteria

Studies meeting the following criteria were excluded from the review:
No comparator group (e.g., single-arm case series).
Studies where oedema is caused by conditions other than nephrotic syndrome, e.g., cirrhosis.
Studies where paediatric data is not separable from adults.
Studies on animal subjects.
Studies published in languages other than English.
Editorials, reviews, commentaries, case reports or case series without a comparator.

Information Sources

We searched the following databases from January 1, 1990, to June 30, 2025: PubMed, Cochrane CENTRAL, Google Scholar and Europe PMC.

Reference lists of eligible articles and previous systematic reviews were manually screened to identify additional studies, although this was deviation from our published protocol but ensured that search was rigorous and no eligible studies skipped.

Search Strategy

A comprehensive search strategy using a combination of MeSH terms and free-text keywords was developed. The full search strategy was published with the protocol on PROSPERO and is summarised in the Appendices.

Study Selection

Search results were imported into Rayyan (Rayyan Systems Inc., Cambridge, MA) for deduplication and screening. Two reviewers independently and blindly screened titles and abstracts for relevance. Conflicts were resolved by consensus, and the results were tracked (Appendices). Full texts were then assessed for eligibility based on the predefined criteria independently by both authors. Disagreements were resolved through discussion and consensus.

Data Extraction

A standardised data extraction form was used to collect information on study characteristics (design, setting and sample size), population demographics, albumin dosing and administration protocols, comparator interventions and outcomes. Two reviewers independently extracted data, and discrepancies were resolved by consensus.

Risk of Bias Assessment

For included studies, we assessed risk of bias using the appropriate tool based on study design. Included RCT was assessed using the Cochrane Risk of Bias 2.0 tool (ROB 2), and observational studies, if included, would have been assessed using ROBINS-I.

Data Synthesis

Due to the limited number of eligible studies and marked heterogeneity in study design and outcome reporting, meta-analysis was not feasible. Results from the included comparative study are reported descriptively.

Mapping Extension (Hybrid Layer)

Given the paucity of fully eligible studies, we extended the review to incorporate a structured evidence mapping exercise aimed at identifying excluded-but-relevant studies that could contribute contextual insight or highlight important evidence gaps. This was a planned deviation from the registered protocol, intended to support hypothesis generation and inform future research priorities. Relevance was defined a priori as studies involving children with nephrotic syndrome who received intravenous albumin, either with or without a comparator, regardless of study design, language or outcomes reported. Studies conducted exclusively in adults but otherwise meeting all inclusion criteria were also included to provide contextual breadth. Candidate studies were identified based on proximity to eligibility criteria, such as absence of a comparator arm, use of non-standard outcomes or inability to isolate paediatric data. All mapped studies were categorised into one of five predefined exclusion domains: (A) inappropriate comparator, (B) no comparator, (C) non-English language, (D) contextual or case series and (E) systematic reviews. Selection of studies for mapping was conducted after final exclusions were logged. The process was performed by one reviewer (SBN) and cross-checked narratively with the screening log by a second reviewer (MAZ). These studies were included in the mapping tables to illustrate key exclusion patterns and signal potential directions for future research.

Results

Systematic Review Findings

A comprehensive search of multiple databases yielded a substantial number of records. After deduplication, all remaining titles and abstracts were screened against predefined eligibility criteria. The majority of exclusions at this stage were due to clearly irrelevant populations (e.g., adults), interventions (e.g., non-albumin agents) or study types (e.g., reviews or case reports), as shown in Table 3.

**Table 3:** Title and abstract screening stage *Total number (705) exceeds excluded articles (550) due to multiple reasons tagged for the same article.

Reason for exclusion	Number of articles*
Wrong study design	284
Wrong intervention	193
Wrong outcome	100
Wrong population	84
Wrong publication type	44

**Figure 1:**
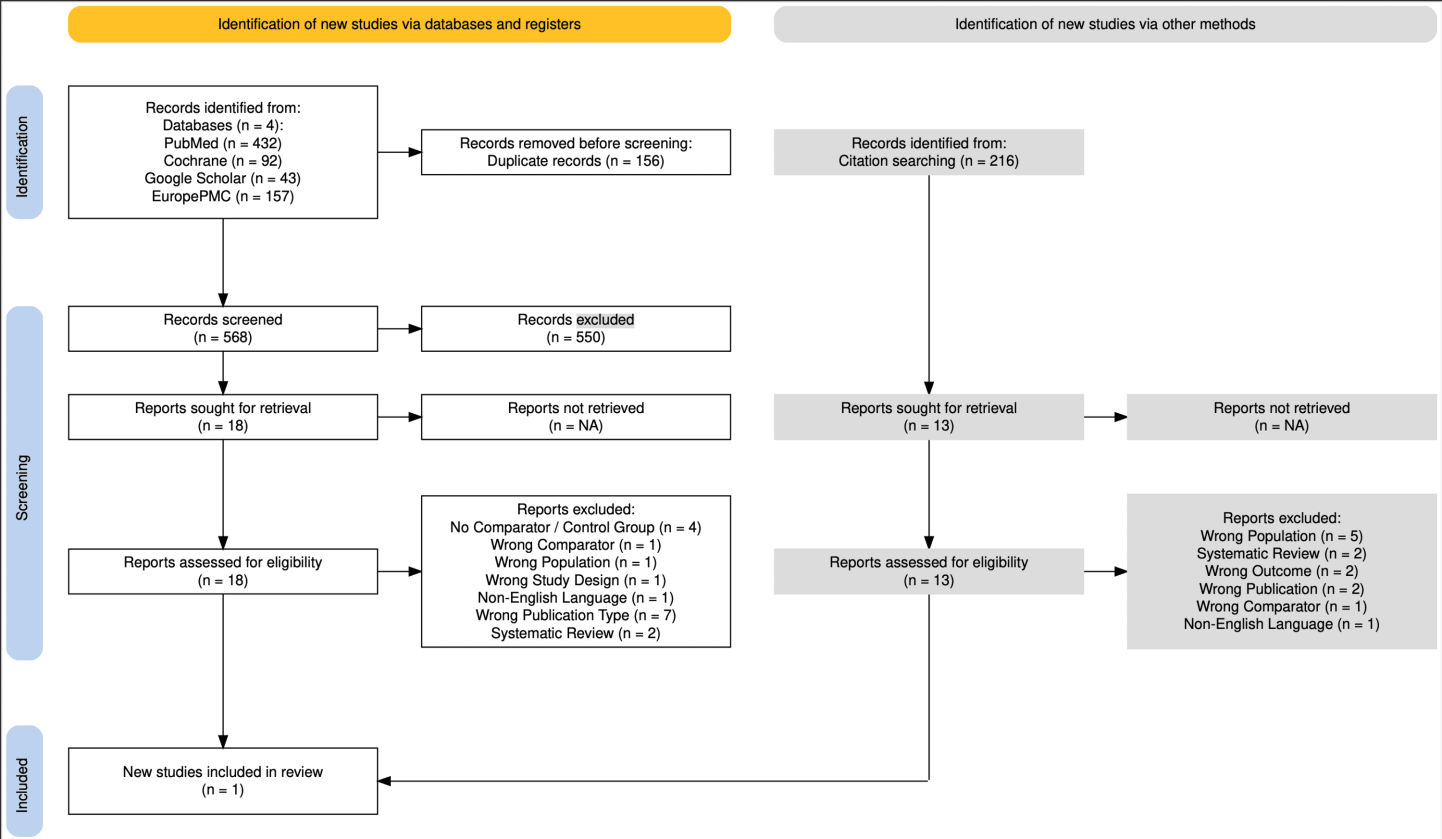
PRISMA flow diagram Created according to PRISMA 2020 Guidelines using the PRISMA creation tool: Creative Commons Attribution (CC BY 4.0) license [15] PRISMA: Preferred Reporting Items for Systematic Reviews and Meta-Analyses

Full-text review was conducted for studies included in the initial screening. Several were excluded at this stage for reasons such as lack of a comparator group or outcomes unrelated to our study question. Ultimately, only one study (Dharmaraj et al. (2009) [11]) met all eligibility criteria and was included in the systematic review. Figure 1 presents the PRISMA flow diagram.

The included study was an open-label, randomised crossover trial conducted in India, comparing IV albumin plus furosemide to furosemide alone in children with nephrotic syndrome and persistent oedema. Sixteen children aged 3-18 years were enrolled and received both interventions in sequence, separated by a 48-hour washout period. Key characteristics of the study design, population, intervention and outcomes are summarised in Table 4.

**Table 4:** Characteristics of the included study Dharmaraj et al. (2009) [11]

Feature	Dharmaraj et al. (2009) [11]
Country	India
Study design	Randomised crossover trial
Population	16 children aged 3-18 years with nephrotic syndrome and persistent oedema despite diuretic therapy
Intervention	20% human albumin infusion followed by furosemide, received by 14 patients
Comparator	Furosemide infusion alone, received by 13 patients
Primary outcomes	Change in urine output and urine sodium excretion
Secondary outcomes	Weight loss, change in urine osmolality, potassium and chloride excretion, osmolal clearance and free water clearance
Notes	Washout period: 48 hours between intervention phases, duration of each phase: 24-hour treatment + 48-hour washout, blinding: not blinded (open label), dropouts and deviations: two children excluded from the furosemide-only phase, three from the albumin + furosemide phase due to early recovery or adverse effects

The primary outcome (change in urine output) favoured the albumin group, with a statistically significant increase compared to furosemide alone (p=0.01 for absolute rates and p=0.008 for percentage change in urine volume from baseline). For the primary outcome of weight loss as per our review question, this study reported a statistically significant difference (p=0.006), favouring the albumin group.

The study reported statistically significant improvements in several urinary parameters favouring the albumin plus furosemide group when measured as percentage change from the baseline. These included greater urine volume, higher urinary sodium, potassium and chloride excretion, increased urine osmolality and osmolal clearance. Blood pressure and serum electrolytes did not differ significantly between groups. The authors concluded that albumin may transiently enhance the diuretic response to furosemide in children with nephrotic syndrome and persistent or refractory oedema resulting in better weight loss, urine output and natriuresis but highlighted that the benefit was likely due to enhanced drug delivery rather than sustained intravascular volume expansion. Table 5 summarises the results of the included study.

**Table 5:** Results of the included study Dharmaraj et al. (2009) [11] Cosm: osmolal clearance ((urine osmolality × urine volume) / plasma osmolality), Cfw: free water clearance ((urine volume - osmolal clearance))

Outcome	Furosemide infusion (FU) (n=13)	Albumin + furosemide infusion (HA+FU) (n=14)	p-value	Statistical significance
Weight loss (%)	0.8 (-1.9 to 4.1)	5.2 (3.1-8.8)	0.006	Yes
Urine volume (mL/kg per hour)	1.33 (0.79-1.88)	3.27 (2.04-4.50)	0.01	Yes
Urine osmolality (mOsm/kg)	368 (318-446)	315 (220-426)	0.13	No
Urine sodium (mEq/day)	30 (10-122)	58 (30-366)	0.08	No
Urine chloride (mEq/day)	56 (16-168)	104 (30-303)	0.07	No
Urine potassium (mEq/day)	14.9 (5-20.5)	30 (11-40.5)	0.07	No
Cosm (mL/day)	880 (510-2105)	1600 (916-4140)	0.01	Yes
Cfw (mL/day)	-162 (-446 to -70)	-190 (-960 to 280)	0.18	No
Adverse events	Metabolic alkalosis (n=1), peritonitis (n=1)	Hypokalaemia (n=1), oedema resolved early (n=2)	-	-

**Figure 2:**
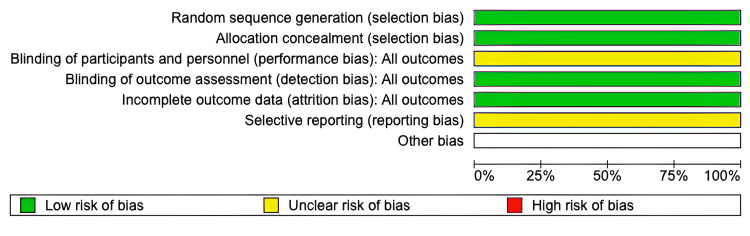
Cochrane ROB 2 tool

These findings, while suggestive of a potential benefit, must be interpreted with caution due to methodological limitations. The study employed a 48-hour washout period, which is likely insufficient to eliminate residual pharmacological effects of albumin and furosemide, thereby introducing a serious risk of carryover bias. In addition, the trial was open-label, and the details provided regarding sequence generation or allocation concealment are minimal. These factors collectively limit the internal validity of the findings. A structured risk of bias assessment using the Cochrane ROB 2 tool judged the study as having “some concerns” overall, due to the open-label design, inadequate washout period and no publication of pre-designed protocol and outcome selection (Figure 2).

We assessed the certainty of evidence for the reported outcomes using the Grading of Recommendations, Assessment, Development and Evaluation (GRADE) approach. As only one randomised trial contributed data, all outcomes began with a high certainty rating but were subsequently downgraded for risk of bias, imprecision, suspected publication bias, inconsistency and indirectness (Table 6). However, the application of the GRADE framework was constrained by the inclusion of only one study. Inconsistencies in reported statistics, absence of confidence intervals for several outcomes and discrepancies between absolute and percentage changes (e.g., in potassium levels) further limited the ability to apply the downgrading criteria with full rigour.

**Table 6:** GRADE summary of findings GRADE: Grading of Recommendations, Assessment, Development and Evaluation Cosm (Osmolal Clearance) = (Urine Osmolality x Urine Volume) / Plasma Osmolality

Outcome	Certainty of evidence (GRADE)	Explanation
Weight loss (%)	⊕⊕⊝⊝ Low	Downgraded for risk of bias (no blinding, carryover bias and lack of published protocol), imprecision (wide CI) and publication bias (single included study)
Urine volume (mL/kg per hour)	⊕⊕⊕⊝ Moderate	Downgraded for risk of bias and publication bias
Urine osmolality (mOsm/kg)	⊕⊝⊝⊝ Very low	Downgraded for risk of bias, imprecision (CI include 0), publication bias and indirectness; graphs show fluctuation without a clear trend or benefit
Urine sodium (mEq/day)	⊕⊝⊝⊝ Very low	Downgraded for risk of bias, imprecision (CI include 0), publication bias and indirectness
Urine chloride (mEq/day)	⊕⊝⊝⊝ Very low	Downgraded for risk of bias, imprecision (CI include 0), publication bias and indirectness (clinical importance unclear)
Urine potassium (mEq/day)	⊕⊝⊝⊝ Very low	Downgraded for risk of bias, imprecision (CI include 0), publication bias, indirectness and inconsistency
Cosm (mL/day)	⊕⊕⊝⊝ Low	Downgraded for risk of bias, imprecision (wide CI) and publication bias (single included study)

Narrative Mapping and Evidence Gap Findings

While only one study met eligibility criteria for full inclusion, several additional studies were considered relevant to the research question and were mapped narratively to describe the broader evidence landscape. These studies were excluded due to methodological limitations, inappropriate comparators, lack of control groups or language restrictions. Their inclusion here highlights critical evidence gaps and patterns across study types (Tables 7, 8).

**Table 7:** Characteristics of studies mapped for evidence gap FFP: fresh frozen plasma, GFR: glomerular filtration rate, RCT: randomised controlled trial

Author (year)	Study design	Population	Intervention and comparator	Outcomes
Huque et al. (2014) [2]	Descriptive cross-sectional study	40 children with nephrotic syndrome and resistant oedema	Albumin + furosemide versus mannitol + furosemide	Weight, oedema, urine output
Singh et al. (2015) [16]	Prospective comparative study	54 children with idiopathic nephrotic syndrome	Albumin versus FFP	Time to oedema resolution and cost of treatment
Bircan et al. (2001) [17]	Prospective observational study	14 children with nephrotic syndrome	Albumin + furosemide (no comparator)	Plasma volume and similar parameters pre- and post-treatment
Garg et al. (2020) [18]	Randomised crossover trial	24 children with nephrotic syndrome	20% albumin versus 5% albumin	Weight reduction, urine output, safety parameters
Akcicek et al. (1995) [19]	Randomised crossover trial	12 adults with nephrotic syndrome	Furosemide versus albumin versus furosemide + albumin combined	Urine output, Na/K excretion, serum albumin
Na et al. (2001) [20]	Randomised crossover trial	7 adults with nephrotic syndrome	Furosemide alone versus albumin + furosemide	Urine output, sodium excretion, diuretic response
Tabel et al. (2008) [21]	Case series	18 children with nephrotic syndrome	Observational study	Observational study
Tsuruga et al. (2009) [22]	Report of case series	6 children with nephrotic syndrome	Recombinant albumin (no comparator)	Safety, allergic reactions, diuretic response
Ghafari et al. (2011) [10]	Randomised control trial	10 adults with nephrotic syndrome	Furosemide versus albumin versus furosemide + albumin combined	Urine output, excretion of sodium, albumin and furosemide
Fliser et al. (1999) [8]	Randomised crossover trial	6 adults with nephrotic syndrome	Furosemide versus albumin versus furosemide + albumin combined	Urine output, sodium balance, GFR
Pasini et al. (2015) [23]	Retrospective observational study	231 children with nephrotic syndrome	Observational study on steroid use	Treatment trends, practice variation
Tanzi et al. (2003) [9]	Drug use evaluation	1649 adult and 23 paediatric inpatients receiving albumin	Audit of albumin use (observational)	Appropriateness of albumin prescriptions
Otukesh et al. (2024) [24]	Quasi-randomised	42 children with nephrotic syndrome	Albumins versus mannitol	Weight reduction
Lee et al. (2000) [25]	Parallel RCT	26 children with nephrotic syndrome	Albumin versus placebo	Weight loss, time to remission, serum electrolytes
Haws and Baum (1993) [13]	Paediatric case series	21 children with nephrotic syndrome	Albumin + 1 diuretic versus albumin + 2 diuretics	Urine output, weight loss

**Table 8:** Excluded studies with reasons for exclusion and evidence mapping FFP: fresh frozen plasma, RCT: randomised controlled trial

Author (year)	Reason for exclusion	Relevance to evidence gap
Huque et al. (2014) [2]	Used mannitol rather than placebo or standard care	Explores albumin efficacy and safety and real-world alternative comparators
Singh et al. (2015) [16]	Comparator not eligible (used FFP)	Supports albumin safety, clinical equipoise and need for placebo-controlled trials
Bircan et al. (2001) [17]	No comparator, observational study	Demonstrates the mechanism of action and the need for RCTs
Garg et al. (2020) [18]	No albumin-free control arm	Explores albimun’s dosing options and relativistic effects
Akcicek et al. (1995) [19]	Adult population	Claims to be the first study to explore the pharmacokinetics of combined albumin + furosemide
Na et al. (2001) [20]	Adult population	Demonstrates enhanced diuresis with albumin and foundational physiological response
Tabel et al. (2008) [21]	Observational study	Explores volume status during oedema of nephrotic syndrome and negates the need for albumin
Tsuruga et al. (2009) [22]	No comparator group	Explores novel albumin formulations and early paediatric safety profile
Ghafari et al. (2011) [10]	Adult population	Supports rationale for albumin co-administration
Fliser et al. (1999) [8]	Adult population	Highlights the transient benefit of albumin and the place for higher diuretic doses
Pasini et al. (2015) [23]	Study question not relevant to our review	Illustrates variation in real-world practice and the need for standardised protocols
Tanzi et al. (2003) [9]	Audit, not interventional or comparative	Reveals widespread off-label albumin use, supports the need for clearer paediatric guidelines
Otukesh et al. (2024) [24]	Foreign language and ineligible comparator	Explores albumin efficacy and safety and real-world alternative comparators
Lee et al. (2000) [25]	Foreign language	Explores the exact question as our review
Haws and Baum (1993) [13]	Retrospective observational study with albumin in all groups	Early observational data demonstrating albumin use and a poor safety profile

Comparator not suitable to assess efficacy of albumin: Two studies used comparators that were active agents with osmotic or diuretic properties, rendering them unsuitable as controls to isolate albumin’s effect. Singh et al. (2015) compared albumin to fresh frozen plasma (FFP) in a randomised trial of 54 children, with both groups receiving furosemide. The study was excluded because FFP itself is an albumin analogue and pharmacologically active, limiting interpretability [16]. Huque et al. (2014) compared albumin-furosemide with mannitol-furosemide in 40 children [2]. No statistically significant difference was observed between groups. The study was excluded because mannitol is an osmotic diuretic and thus an inappropriate comparator for assessing albumin’s efficacy in isolation.

No comparator: Two studies used albumin in all treatment arms or as part of an uncontrolled intervention, precluding comparative conclusions. Both studies demonstrated feasibility but could not inform on effectiveness. Garg et al. (2020) retrospectively studied 25 children who all received albumin plus furosemide, with differences in dosing protocols; hence, the study was excluded [18]. Bircan et al. (2001) evaluated albumin plus diuretics in children with minimal change nephrotic syndrome, but the study was excluded for not having a comparator arm, and the outcomes did not align with our research question either [17].

Language of publication: Two studies met some of the other eligibility criteria but were excluded due to non-English publication. Otukesh et al. (2004), published in Persian, compared albumin and mannitol, showing significant differences for some outcomes, while none for the other [24]. Lee et al. (2000), the only included study in the Cochrane review by Ho et al. (2019), was a Korean-language single-blind RCT comparing albumin with furosemide versus furosemide alone in a group of 26 patients [25]. It showed a shorter time to remission in the albumin group, but an earlier time of first relapse as well. There was no significant difference in relapse rate at one year and relapse frequency within the first year. These studies underscore the risk of language bias in evidence synthesis.

**Figure 3:**
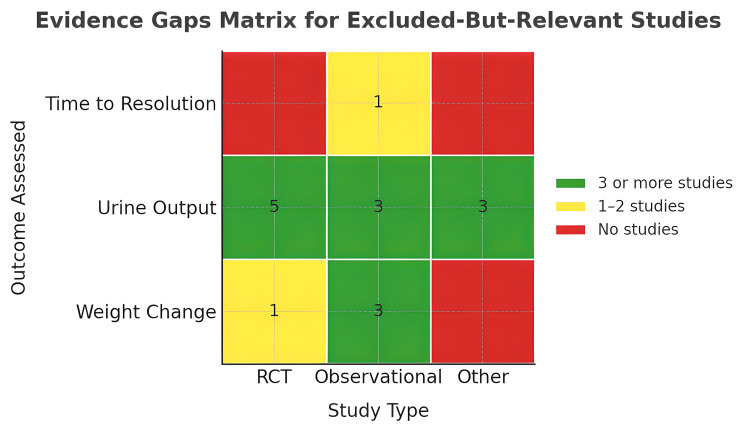
Outcomes reported by different study types among excluded studies RCT: randomised controlled trial

Contextual but ineligible for mapping: Nine studies were excluded due to population mismatch, non-comparative designs or focus on biochemical/pharmacokinetic outcomes (Figure 3). Fliser et al. (1999) [8], Ghafari et al. (2011) [10] and Na et al. (2001) [20] studied adult or mixed populations. Pasini et al. (2015) [23] and Tanzi et al. (2003) [9] reviewed the practices across various paediatric units. Tabel et al. (2008) [21] lacked a comparator, while Haws and Baum [13] (1993) was a retrospective observational study. Akcicek et al. (1995) [19] and Tsuruga et al. [22] (2009) were focused on albumin kinetics.

Systematic reviews: Four previous reviews were identified that addressed the use of albumin in patients with oedema or hypalbuminaemia. None were eligible for formal inclusion as per our protocol. Elwell et al. (2003) [7] and Kitsios et al. (2014) [14] synthesised evidence primarily from adult populations in critical care or cirrhotic settings, without a specific focus on nephrotic syndrome or children. Hedin et al. (2022) did include one paediatric nephrotic syndrome study, but otherwise drew mainly on adult trials and did not stratify outcomes by age group [3]. Ho et al. (2019), a Cochrane review, aimed to assess the effects of albumin infusion in people with nephrotic syndrome [5]. Although the scope included both adults and children, only one eligible study (Lee et al. (2000) [25]) was identified and included. This study involved the paediatric population but was published in Korean and thus was excluded from our review due to language restrictions.

**Figure 4:**
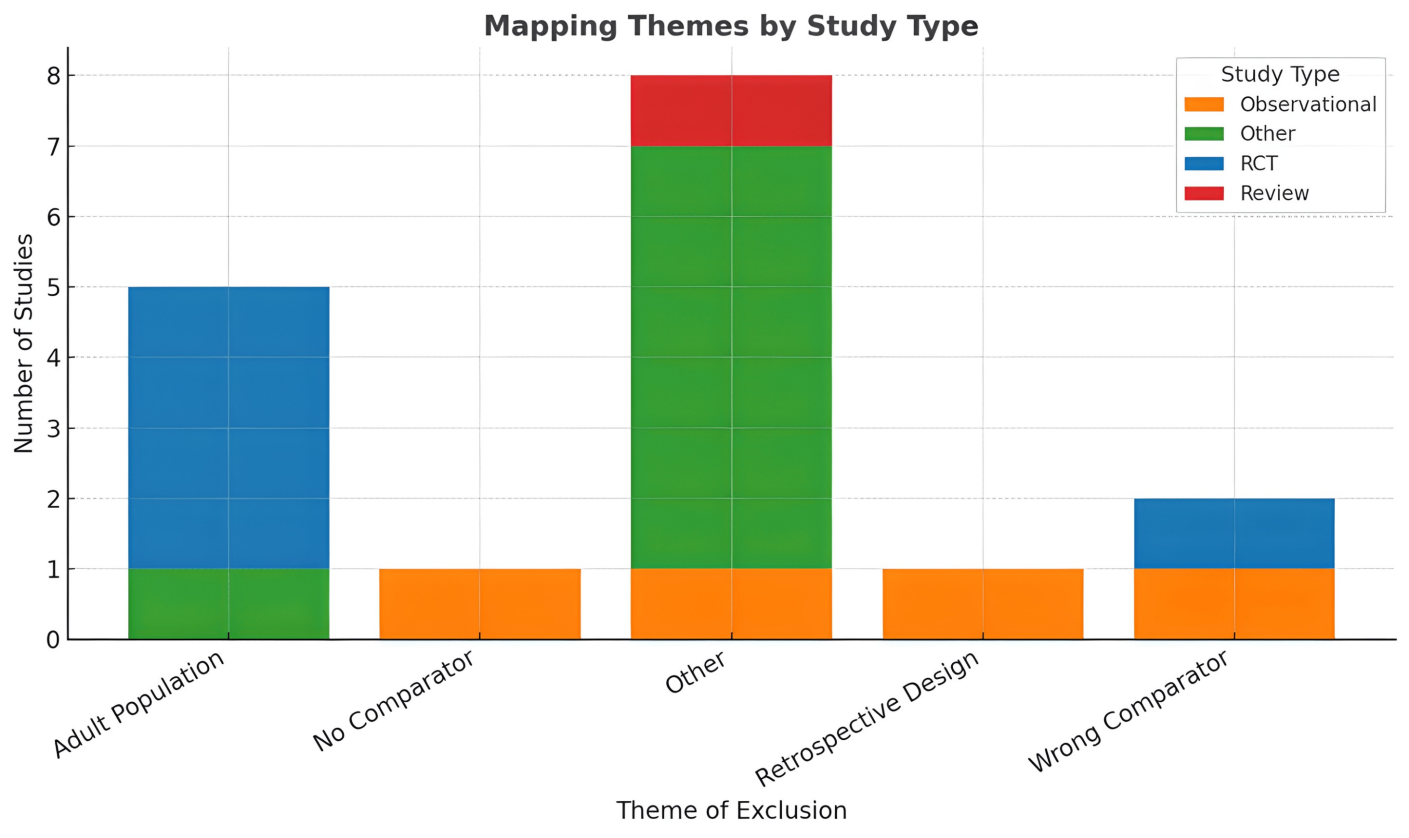
Ineligibility mapped by study types RCT: randomised controlled trial

Together, these reviews highlight a critical gap in the evidence base: the near-complete absence of high-quality randomised trials evaluating intravenous albumin in children with nephrotic syndrome. They also demonstrate inconsistent population definitions, lack of paediatric subgroup analysis and variation in outcome reporting, all of which contribute to uncertainty about when and how albumin should be used in this setting (Figure 4).

Discussion

Summary of Main Findings

This systematic review identified only one randomised controlled trial (Dharmaraj et al. 2009 [11]) that met full inclusion criteria for assessing the effectiveness and safety of intravenous albumin in hospitalised children with nephrotic syndrome and oedema. In Dharmaraj et al. (2009), a crossover trial, the combination of 20% albumin and intravenous furosemide produced a statistically significant increase in urine output and weight loss compared to furosemide alone [11]. Secondary outcomes, including changes in urine sodium, potassium, osmolality and osmolal clearance, also significantly improved in the albumin + furosemide arm. However, these changes were transient, with peak effects observed within the first few hours, and no significant difference in the time to oedema resolution or in reported adverse events between arms. These results suggest short-term physiological benefits without strong evidence of sustained clinical outcomes. The neutral tone of the findings aligns with the study’s limited sample size and lack of blinding, necessitating cautious interpretation.

Interpretation of Evidence

The findings from Dharmaraj et al. (2009) support the hypothesis that albumin may augment the natriuretic and diuretic response to loop diuretics in hypoalbuminemia states [11]. Mechanistically, this is consistent with pharmacokinetic principles suggesting that albumin enhances furosemide delivery to its site of action in the nephron [7,14,26]. Albumin-bound furosemide is secreted into the proximal tubule via organic anion transporters, and hypoalbuminaemia may limit this process by increasing volume of distribution and decreasing renal delivery. Albumin infusion may transiently increase intravascular volume and perfusion, improving drug delivery [17].

However, the study by Dharmaraj et al. has notable limitations that temper interpretation. Its open-label design introduces performance and detection bias. The crossover nature raises concerns about carryover effects, although a 48-hour washout period was used. Furthermore, the small sample size (n=16), with further exclusions during the interventions, limits precision and generalisability. Importantly, while short-term urine output and electrolyte changes were documented, patient-centred outcomes such as hospital stay, symptom relief or time to resolution were not measured.

Evidence Mapping Insights

Our mapping exercise of excluded-but-relevant studies revealed several recurring themes that shed light on the fragmented nature of the current evidence base. A substantial proportion of studies employed uncontrolled, single-arm designs or lacked an appropriate comparator group altogether, making it difficult to isolate the effect of albumin from natural diuretic response or disease progression (e.g., Bircan et al. (2001) [17], Huque et al. (2014) [2] and Singh et al. (2015) [16]). Among studies that did include comparison arms, many were excluded due to mismatched interventions or use of non-standardised co-therapies, reflecting significant heterogeneity in study design. Language barriers also emerged as a noteworthy issue: relevant studies such as Lee et al. (2000) [25] and Otukesh et al. (2024) [24], published in Korean and Persian, respectively, could not be included, highlighting the ongoing challenge of evidence exclusion based on language rather than content.

Another consistent finding was the prevalence of mixed populations or adult-only cohorts (e.g., Akcicek et al. (1995) [19], Ghafari et al. (2011) [10] and Fliser et al. (1999) [8]), which limits the applicability of findings to paediatric nephrotic syndrome. Even when children were included, subgroup data were often not disaggregated, further reducing interpretability. Outcome heterogeneity posed an additional barrier to synthesis. Across the mapped studies, researchers variably measured weight loss, urine volume, serum albumin levels, fractional excretion of sodium, time to oedema resolution or plasma volume expansion, without consistent timing, units or definitions. Safety assessment was also poorly addressed: few studies systematically reported adverse events, volume overload or electrolyte disturbances, and definitions of “refractory oedema,” “severe hypoalbuminaemia” or “response to treatment” were either absent or used inconsistently.

Despite these limitations, the mapping process provided valuable insights into clinical practice patterns and evidence gaps. The persistent use of albumin in some studies despite weak or equivocal data suggests clinical equipoise remains unresolved. In particular, the concentration of studies around early diuretic response reflects a mechanistic focus, often decoupled from long-term patient-centred outcomes. Repeated albumin dosing, cost implications, discharge timing and quality of life remain largely unexplored in the mapped literature. Collectively, these mapped studies highlight the lack of standardised methodologies, uniform outcome reporting and paediatric-specific trials, reinforcing the urgent need for well-designed prospective studies that address these deficiencies.

Comparison With Previous Reviews

Prior reviews and meta-analyses have repeatedly examined the role of albumin in patients with hypoalbuminaemia and diuretic resistance, yet they have consistently arrived at low-certainty conclusions. Elwell et al. (2003) conducted a narrative review focusing on the pharmacological rationale for combining albumin with furosemide in diuretic-resistant oedema [7]. They suggested that while some small studies showed transient clinical benefit, particularly in nephrotic syndrome, the overall evidence was inconsistent, heterogeneous and limited in quality. Their review primarily emphasised theoretical mechanisms such as improved intravascular volume status and enhanced diuretic delivery, rather than robust clinical outcomes. This mechanistic lens closely parallels some of the rationale presented in our review, but their conclusions were largely speculative due to the absence of systematic methodology or paediatric stratification.

Kitsios et al. (2014) performed a more methodologically rigorous meta-analysis, including eight RCTs, the majority of which involved adult populations and used crossover or parallel trial designs [14]. They found statistically significant increases in urine output and sodium excretion within eight hours of albumin administration, but these effects did not persist beyond 24 hours. Importantly, they reported no significant benefits in terms of patient-centred outcomes such as symptom resolution, length of stay or need for further intervention. Their findings reinforce the notion of a short-term physiological benefit that may not translate into meaningful clinical improvement, an observation echoed by the included study in our review (Dharmaraj et al. (2009) [11]).

Ho et al. (2019) conducted a Cochrane systematic review specifically evaluating albumin for the treatment of oedema in nephrotic syndrome [5]. Their review was comprehensive in scope but identified only one eligible study (Lee et al. (2000) [25]) published in Korean, which limited their ability to perform meta-analysis or draw strong conclusions. They highlighted pervasive methodological limitations across available studies, including unclear definitions of outcomes, small sample sizes and poor reporting. Their ultimate conclusion, that there was insufficient evidence to recommend routine albumin use in nephrotic syndrome, remains in alignment with our findings, although their inclusion of a non-English language study diverges from our eligibility criteria.

Hedin et al. (2022) represent the most recent and expansive attempt to synthesise the evidence base, incorporating both paediatric and adult populations [3]. They reported some increases in urine output, particularly with early combination therapy, but noted inconsistent effects on sodium excretion, no clear trends in long-term outcomes and a lack of stratified analysis by age group. Although one paediatric study was included, the review did not differentiate paediatric findings from adult results in a meaningful way, limiting its direct applicability to child health contexts.

Across all these reviews, a consistent pattern emerges: transient improvements in physiological parameters, no sustained effect on patient-centred outcomes and methodological weaknesses that preclude strong recommendations. Despite this, albumin continues to be used in practice, raising important questions about the drivers of clinical behaviour. These may include institutional norms, clinician perceptions of benefit in specific subgroups (e.g., those with severe hypoalbuminaemia or refractory oedema), lack of alternative therapies or a risk-averse approach in complex cases. None of the previous reviews have directly addressed this disconnection between guideline uncertainty and real-world use.

Our review, while similarly constrained by the limited evidence base, offers unique contributions by focusing specifically on hospitalised children with nephrotic syndrome and clinically significant oedema. Furthermore, our structured evidence mapping of excluded studies sheds additional light on the landscape of partially relevant research and highlights specific gaps that future studies must address. While our conclusions are aligned with the cautionary stance of earlier reviews, our methodological approach aims to move the field closer to resolving the enduring uncertainty surrounding albumin use in this vulnerable population.

Strengths of This Review

Table 9 shows the strengths of our systematic review.

**Table 9:** Strengths of this review PROSPERO: International Prospective Register of Systematic Reviews, PRISMA: Preferred Reporting Items for Systematic Reviews and Meta-Analyses

Category	Details
Pre-registered protocol	The review was registered on PROSPERO prior to data extraction, enhancing transparency and reducing bias.
PRISMA adherence	All reporting followed PRISMA 2020 guidelines, including study selection flow diagrams and structured methods.
Hybrid methodology	We combined rigorous systematic review with narrative evidence mapping, allowing both synthesis and exploration of excluded-but-relevant literature.
Paediatric focus	Exclusively focused on children aged 1-18 years with nephrotic syndrome, improving clinical applicability.
Dual reviewer screening	Title/abstract and full-text screening were conducted independently by two reviewers to reduce selection bias.
Comprehensive literature base	We included peer-reviewed studies and prior systematic reviews, with additional full-text articles identified through snowballing and reference list screening, substantially expanding the evidence base beyond initial database results.

Limitations of This Review

Table 10 shows the limitations of this review.

**Table 10:** Limitations of this review RCT: randomised controlled trial

Category	Details
Single included RCT	Only one trial met full inclusion, limiting quantitative synthesis and meta-analysis.
Small sample size	The included RCT had only 16 participants, increasing imprecision and reducing generalisability.
Heterogeneous excluded studies	Excluded-but-relevant studies varied in design, outcomes and population, limiting comparability.
Language bias	Non-English studies without accessible full texts were excluded, which may omit valuable data.
Surrogate outcomes dominate	Most studies focused on physiological rather than patient-centred outcomes.
Short follow-up periods	Few studies assessed outcomes beyond 24-48 hours, limiting understanding of sustained effects.
Pragmatic mapping inclusion	Decisions to map rather than formally include certain studies may introduce indirectness.
Protocol deviation I	We adopted a hybrid approach by adding narrative evidence mapping layer, which was not originally planned. The study title was also amended to reflect this broader scope.
Protocol deviation II	Although our protocol did not plan for snowballing or reference screening, these methods were employed post hoc to identify additional relevant studies.

Clinical Implications

Based on available evidence, routine use of intravenous albumin in all hospitalised children with nephrotic syndrome and oedema is not supported. The combination may be considered in select cases, particularly those with severe hypoalbuminaemia (e.g., serum albumin < 20 g/L), refractory oedema unresponsive to escalating diuretics and no contraindications to fluid expansion (e.g., absence of pulmonary congestion or cardiac dysfunction). Even in these cases, cautious monitoring of fluid status, electrolytes and renal function is essential. Alternative strategies such as mannitol-furosemide or sequential nephron blockade (e.g., thiazide + loop diuretics) may offer comparable benefit with potentially lower risk [2,27].

Directions for Future Research

Adequately powered, blinded RCTs should be conducted in children with nephrotic syndrome to evaluate the efficacy and safety of albumin + diuretic therapy. Standardised definitions for “diuretic resistance,” “hypoalbuminaemia” and “oedema resolution” need to be used. We also recommend comparing albumin + furosemide versus alternative strategies (e.g., mannitol, hypertonic saline or combination diuretics). Long-term outcomes, such as hospital length of stay, relapse rates, time to remission and quality of life, must be incorporated. Mandatory adverse event reporting, including cardiovascular outcomes, fluid overload and electrolyte disturbances, is essential. For low-resource settings, where albumin is expensive or scarce, cost-effectiveness analyses must be conducted. Lastly, it is necessary to develop consensus guidelines based on available evidence to guide albumin use in paediatric nephrotic syndrome.

## Conclusions

Current evidence for intravenous albumin in hospitalised children with nephrotic syndrome is very limited. The single included trial shows transient improvements in urine output and weight, but patient-centred outcomes remain largely unassessed, and benefits are modest and short-lived. Narrative mapping and prior reviews highlight major evidence gaps, including heterogeneous populations, varied interventions, absent comparators and sparse safety data. Routine albumin use cannot be universally recommended; it may be considered only in severe hypoalbuminemia or refractory oedema, with careful monitoring.

There is an urgent need for well-designed, adequately powered paediatric RCTs with standardised outcomes and systematic safety reporting. Future studies should also assess alternative therapies and cost-effectiveness to inform clinical guidelines. In summary, current evidence confirms only modest, short-term effects with no established patient-centred benefits. Major uncertainties remain regarding long-term safety, subgroup responses and broad clinical outcomes. Until these gaps are addressed, use of albumin should be highly cautious and guided by careful risk-benefit assessment.

## Appendices

Table 11 presents the PRISMA 2020 checklist.

**Table 11:** PRISMA 2020 compliance table PRISMA: Preferred Reporting Items for Systematic Reviews and Meta-Analyses, PICOST: Population, Intervention, Comparator, Outcomes, Study Designs and Timeframe, RCT: randomised controlled trial

Item	Description	Location/implementation in the manuscript
1	Title: Identify the report as a systematic review	Title page clearly states “A Systematic Review and Mapping of the Evidence Landscape,” highlighting a hybrid design.
2	Abstract: Structured summary of objectives, methods, results and conclusions	Abstract structured under headings: Background, Objectives, Search Methods, Selection Criteria, Data Collection and Analysis, Results, Authors’ Conclusion. Fully self-contained.
3	Rationale: Describe the rationale for the review	Introduction section: Explains gaps in existing literature, limitations of prior reviews and clinical relevance.
4	Objectives: Explicit statement of objectives	Introduction, final paragraph: Clearly states the aim to evaluate the effectiveness and safety of IV albumin in children and map excluded studies.
5	Eligibility criteria	Methods → Eligibility Criteria: PICOST framework provided; inclusion/exclusion criteria listed in detail.
6	Information sources	Methods → Information Sources: Databases searched (PubMed, Cochrane CENTRAL, Google Scholar and Europe PMC), dates of search and hand-searching of references described.
7	Search strategy	Methods → Search Strategy: Full search strings provided in table: Search strategy; use of MeSH terms, free text, filters and date limits documented.
8	Selection process	Methods → Study Selection: Dual independent screening in Rayyan, conflict resolution process described. PRISMA flowchart included (Figure: PRISMA flow diagram).
9	Data collection process	Methods → Data Extraction: standardised form, dual independent extraction, discrepancy resolution by consensus.
10	Data items	Methods → Data Extraction + tables: population, interventions, comparators, outcomes and study design collected; tables: Characteristics of the included study, Results and GRADE summary of findings.
11	Study risk of bias assessment	Methods → Risk of Bias: Cochrane ROB 2 tool applied to included RCT; narrative description of mapped excluded studies. Figure: ROB 2 tool included.
12	Effect measures	Results → Systematic Review Findings: Reported outcomes with GRADE summary of findings; p-values and statistical significance included in tables.
13	Synthesis methods	Methods → Data Synthesis: Narrative synthesis described; rationale for no meta-analysis due to heterogeneity and single included study explained.
14	Reporting bias assessment	Methods → Risk of Bias + GRADE summary: certainty of evidence downgraded for suspected publication bias, imprecision and indirectness; fully explained in table: GRADE summary of findings. Not enough studies included to compile a funnel plot.
15	Certainty assessment	Results → GRADE summary of findings: certainty (High, Moderate, Low, Very low) reported per outcome with explanatory notes.
16	Study selection	Results → Title and abstract screening conflicts resolution table; PRISMA flow diagram; narrative description of excluded and included studies.
17	Study characteristics	Results → tables: Characteristics of the included study, Mapping rationale for excluded studies; narrative mapping describes additional studies.
18	Risk of bias in studies	Results → figure: Cochrane ROB 2 tool; narrative description for excluded studies.
19	Results of individual studies	Results → table: Results of the included study: Dharmaraj et al. (2009) [11]; key findings described in text.
20	Results of syntheses	Results → narrative synthesis: Short-term physiological effects of albumin summarized; mapping insights highlighted.
21	Reporting biases	Methods → Risk of Bias, GRADE summary; narrative discussion of potential biases including language and publication bias.
22	Certainty of evidence	Results → GRADE summary of findings table, narrative description in text for each outcome.
23a	Discussion - interpretation	Discussion → Summary of Main Findings subheading and Interpretation of Evidence subheading
23b	Discussion - limitations of evidence	Discussion → Interpretation of Evidence subheading
23c	Discussion - limitations of review	Discussion → Limitations of This Review subheading
23d	Discussion - implications	Discussion → Clinical Implications and Future Research: recommendations and research gaps highlighted; neutral tone maintained.
24	Registration and protocol	Methods → protocol registration: PROSPERO ID CRD420251102387 included with URL with amendment updates.
25	Support	Independent reviewers with no source of external funding.
26	Competing interest	Both authors declare no conflict of interest.
27	Availability of data	All relevant data sets published as part of the manuscript, and any additional data will be available from the corresponding author on reasonable request.

Table 12 summarises the full search strategy.

**Table 12:** Search strategy

Database	Search string	Filters applied	Date searched	Results retrieved
PubMed	(“nephrotic syndrome”[MeSH Terms] OR “nephrotic syndrome”) AND (“albumin” OR “intravenous albumin” OR “IV albumin” OR “human albumin” OR “20% albumin”) AND (child* OR paediatric* OR pediatric*)	English, humans, child (birth-18 years), 1990-2025	July 11, 2025	432
Cochrane CENTRAL	(nephrotic syndrome OR nephrotic syndrome) AND (albumin OR intravenous albumin OR IV albumin OR human albumin OR 20% albumin) AND (child OR paediatric OR pediatric)	None	July 11, 2025	4 reviews, 88 trials
Google Scholar	allintitle: albumin nephrotic syndrome children	Year: 1990-2025	July 11, 2025	43
Europe PMC	((TITLE_ABS:(Nephrotic Syndrome) AND TITLE_ABS:(Albumin)) AND TITLE_ABS:(Child OR Pediatric OR Paediatric))	Year: 1990-2025	July 11, 2025	157

Table 13 presents the title and abstract screening conflict resolution.

**Table 13:** Title and abstract screening conflict resolution

Process type	Screening stage	Number of articles	Results (included/excluded/maybe)	Notes
Independent and blind review	Title and abstract screening	568	Included: 9, excluded: 516, conflicts: 43	Articles screened independently by two reviewers
Deliberation and consensus	Marked: maybe versus include	8	Included: 8/excluded: 0	Automatically included all for full-text review
	Marked: maybe versus exclude	24	Included: 0, excluded: 24	All excluded after re-review
	Maybe by both reviewers	6	Included: 0, excluded: 6	All excluded after discussion
	Conflicts marked: include versus exclude	5	Included: 1, excluded: 4	Excluded studies: 1. Setiamal (2024): unrelated intervention and outcomes, 2. Cakirsa (2020): unrelated outcomes in observational study, 3. Toledo (2017): pharmacoeconomics study with unrelated outcomes, 4. Lijuan (2021): foreign language (Mandarin)
